# Effectiveness of Compounded Bioidentical Hormone Replacement Therapy: An Observational Cohort Study

**DOI:** 10.1186/1472-6874-11-27

**Published:** 2011-06-08

**Authors:** Andres D Ruiz, Kelly R Daniels, Jamie C Barner, John J Carson, Christopher R Frei

**Affiliations:** 1 College of Pharmacy, The University of Texas, Austin, TX, University Station A1900, Austin, TX, 78712, USA; 2 Pharmacotherapy Education and Research Center, The University of Texas Health Science Center, San Antonio, TX, 7703 Floyd Curl Dr., MSC-6220, San Antonio, TX, 78229, USA; 3 Oakdell Pharmacy Inc., San Antonio, TX, 7220 Louis Pasteur Dr., Suite 176, San Antonio, TX, 78229, USA

## Abstract

**Background:**

Bioidentical Hormone Replacement Therapy (BHRT) is believed it to be a safer and equally effective alternative to Conventional Hormone Therapy for the relief of menopausal symptoms; however, data are needed to support these claims. The objective of this study is to evaluate the effectiveness of compounded BHRT provided in six community pharmacies.

**Methods:**

This was an observational cohort study of women between the ages of 18-89 who received a compounded BHRT product from January 1, 2003 to April 30, 2010 in six community pharmacies. Data included patient demographics, comorbidities, therapeutic outcomes, and hormone therapies. Women self-rated menopausal symptoms as absent, mild, moderate, or severe. Descriptive statistics were used to characterize the patient population, BHRT use, and adverse events. Patient symptom severity was compared at baseline and 3 to 6 months follow-up using the Wilcoxon signed-rank test.

**Results:**

Women (n = 296) receiving BHRT at Oakdell Pharmacy had a mean (standard deviation) age of 52 (9) years. The most common BHRT dosage forms utilized were topical (71%) and oral (43%). Compounded BHRT regimens were generally initiated at low doses regardless of route. Women experienced a 25% decrease in emotional lability (p < 0.01), a 25% decrease in irritability (p < 0.01), and a 22% reduction in anxiety (p = 0.01) within 3 to 6 months. These women also experienced a 14% reduction in night sweats (p = 0.09) and a 6% reduction in hot flashes (p = 0.50).

**Conclusions:**

This study demonstrates that compounded BHRT improves mood symptoms. Larger studies are needed to examine the impact on vasomotor symptoms, myocardial infarction and breast cancer.

## Background

Bioidentical Hormone Replacement Therapy (BHRT) describes supplementation of hormones that are molecularly identical to those hormones produced in the human body. Unlike manufactured Conventional Hormone Therapy (CHT), such as conjugated estrogens (CE) and medroxyprogesterone acetate (MPA), BHRT hormones do not contain extra structural moieties which may alter hormone receptor binding and function in the human body.

The Professional Compounding Centers of America (PCCA) estimates that 1 in 4 compounded products in the United States are a form of Hormone Replacement Therapy (HRT) [1]. Compounded BHRT is a form of personalized medicine whereby the dose, regimen, and dosage forms are customized based on the patient's symptoms, hormone levels, and preferences. Hormone levels are obtained by either serum or salivary testing and are measured to assess a woman's hormonal stage of life and to create customized therapies. The idea is that these individualized preparations containing bioidentical hormones may improve the safety and effectiveness of treating menopausal symptoms. Compounded BHRT is perceived to be more effective, better tolerated, with fewer health risks than its CHT counterpart [2-4].

This study first describes the characteristics and prescribing patterns of compounded BHRT in six community pharmacies. Three specific aims are then addressed: (1) determine if compounded BHRT is effective in treating menopausal symptoms, (2) identify which BHRT compounds are most effective, and (3) determine if compounded BHRT is safe.

## Methods

This was an observational cohort study of female patients age 18-89 treated for menopause-related hormone imbalances from January 1, 2003 to April 30, 2010 in six community pharmacies. The University of Texas Health Science Center at San Antonio Institutional Review Board reviewed and approved this study; protocol number HSC20090507H. Oakdell Pharmacy, Inc. is comprised of six independent community pharmacies located throughout San Antonio, Texas and has offered BHRT consultation services since 2003. Currently, more than 1,000 women receive their compounded BHRT at Oakdell Pharmacy. Patient charts are maintained locally at the pharmacy.

Pharmacists who provide BHRT consultation services at Oakdell Pharmacy are trained through PCCA educational symposiums and other BHRT-focused seminars. At these seminars, credentialed medical professionals (e.g., physicians and pharmacists) provide evidence-based BHRT education. In addition, pharmacists who care for BHRT patients at Oakdell must have BHRT-related continuing education credit.

Patients are referred to the Oakdell Wellness Center through physicians, family members, and friends. BHRT consultation services consist of an extensive initial evaluation, hormone replacement education, and follow-up visits. Patients complete a new patient evaluation form and a laboratory hormone panel prior to their initial visit. This hormone panel, determined through serum or salivary testing, aids in identification of sex hormone deficiencies, adrenal function deficiencies, and thyroid dysfunction. Additional hormone panels are recommended 3-6 months after BHRT initiation, annually, and when deemed necessary to assist with BHRT customization. During the initial evaluation, patients are questioned regarding medical history, menopausal symptoms, and treatment goals. They are educated on several components of hormone therapy including: hormonal changes of menopause, causative factors associated with menopausal symptoms, risks and benefits of hormone therapy (both conventional and bioidentical therapies), and different BHRT dosage forms. During initial evaluation and follow-up visits, pharmacists use a standardized form to monitor symptom resolution and adverse effects. This form lists several symptoms associated with menopause. Patients are asked to indicate whether their symptoms are "absent," "mild," "moderate," or "severe."

The pharmacist and patient decide on an individualized treatment plan consisting of either (1) a prescription recommendation to be faxed to their physician for modification, signature, and approval and/or (2) over-the-counter (OTC) products. The pharmacist's letter to the physician describes the patient's hormone imbalance(s), laboratory results, and current menopausal symptoms. It also suggests a customized BHRT regimen and outlines the benefits of compounded BHRT. BHRT compounds vary by active ingredients, dose, and dosage form. Once the physician's approval is received, the individualized BHRT compound is prepared. The most common compounded BHRT hormones include: biest (estriol/estradiol in varying concentrations), triest (estriol/estradiol/estrone), progesterone, testosterone, and dehydroepiandosterone (DHEA). Table 1 depicts common bioidentical hormone dose classifications.

**Table 1:** Common Compounded Bioidentical Hormones Dose Classifications

Dose Classification	Dose Range
**Topical Estrogen**	

Low Dose	≤0.5 mg

Moderate Dose	0.51-1 mg

High Dose	>1 mg

**Oral Estrogen**	

Low Dose	≤1 mg

Moderate Dose	1.1-2 mg

High Dose	>2 mg

**Topical Progesterone**	

Low Dose	<20 mg

Moderate Dose	21-50 mg

High Dose	>50 mg

**Oral Progesterone**	

Low Dose	<100

Moderate Dose	101-200

High Dose	>200

Patients are instructed to complete monthly evaluation forms for the first few months and every six months thereafter to monitor menopausal symptoms and side effects. Upon review of a patient's evaluation form, the pharmacist decides if further laboratory work, telephone, or face-to-face follow-up is needed. The pharmacist then makes changes to the patient's therapy in consultation with the patient and their physician.

### Patient Eligibility

Women ≥18 years of age receiving compounded BHRT at Oakdell Pharmacy Inc., from January 1, 2003 to April 30, 2010 were eligible for this study. Patients were excluded if they received compounded BHRT from Oakdell Pharmacy, but were managed outside the Oakdell Pharmacy system (i.e., physicians' offices). Patients were also excluded if they did not complete a follow-up hormone evaluation form. All data were collected at Oakdell Pharmacy from the existing medical records.

### Data Collection

A customized, data collection instrument was developed using Microsoft Access 2007^® ^software. The instrument was designed to mimic the appearance of the initial patient intake and follow-up forms used during pharmacist consultations at Oakdell Pharmacy. All research personnel were trained on the tool prior to collection of any patient data. Data included patient demographics, comorbidities, laboratory values, medications, and adverse effects of hormone therapy. The last-observation-carried-forward (LOCF) method was used to determine BHRT effectiveness at 3 and 6 months follow-up. If patient data were available at 6 months, those data were used. That is, if patient data were available at 3 months, but not 6 months, then the data from 3 months were used. LOCF is an accepted method used by other HRT studies [5-7].

The dependent variables utilized in this study were vasomotor symptoms, mood symptoms, myocardial infarction, and breast cancer. A decrease in symptom intensity was defined as a decrease in symptom ratings from baseline to follow-up (three to six months). Baseline and follow-up symptom ratings were also compared at each time point using the percentage of patients reporting either "moderate" or "severe." Reduction in symptom severity was also evaluated in a subgroup analysis of women between the ages of 40 and 70 years.

Myocardial infarction and breast cancer adverse event rates were reported as events and cases per 10,000 person years. Adverse event (AE) rates were calculated with the following method: number of AE/[(average time to AE) X (# of patients with AE follow-ups). This number was then multiplied by 10,000 to give AE rates per 10,000 person years.

The independent variables utilized in this study were BHRT regimen and BHRT dosage form. The term "estrogen" was used to describe the use of any of the estrogen combinations [i.e., triest (estriol 80%/estradiol 10%/estrone 10%), biest (estriol 80%/estradiol 20%), biest (estriol 70%/estradiol 30%), and biest (estriol 50%/estradiol 50%)].

Patient's length of therapy (LOT) was obtained from the pharmacy records and patient charts. LOT was calculated using the following method: date of most current BHRT prescription fill minus date of original fill. The patient's treatment timeframe was obtained from pharmacy records and was calculated as the date of follow-up minus the date of baseline assessment.

### Data and Statistical Analysis

All statistical analyses were performed using JMP 8.0^® ^(SAS Corp., Cary, NC). Statistical significance was defined as an alpha less than 0.05. Descriptive statistics (e.g., means, medians, and frequencies) were used to characterize the patient demographics, BHRT use, symptom resolution, and adverse effects. Patient demographics, baseline characteristics, comorbid conditions, baseline menopausal symptoms, dosing regimens, symptom improvement, and adverse effects were compared between groups. Categorical variables were compared using chi-square and Fisher's exact tests. Continuous variables were tested for normality using the Shapiro Wilk-W Test. Normally distributed variables were reported as means (standard deviations), while non-normally distributed variables were reported as medians (25th and 75th percentiles). Paired data were compared using the Wilcoxon signed-rank test. All tests were two-sided.

## Results

### Population Characteristics

Of 431 charts reviewed, 296 women met study criteria. The mean (standard deviation) age was 52 (9) years and the mean weight was 153 (32) lbs. Only 1% of women reported a history of heart disease; however, nearly one-quarter of women reported at least one of the following CHD risk factors: dyslipidemia (21%), hypertension (12%), or diabetes (4%). Few women reported a history of thyroid disorders (14%), cancer (3%), or coagulopathies (2%). One-third of the women had a hysterectomy and one-quarter had an oophorectomy. Many women reported caffeine (73%) or alcohol (54%) use, but few reported tobacco (8%) use. The majority of women had not received prior hormone therapy; 8% reported prior CHT use and 13% reported prior BHRT use. When compared to those women who were excluded, women included in this study were more likely to have had a hysterectomy (p = 0.02), previous BHRT use (p < 0.01), and previous CHT use (p < 0.01) at baseline.

### BHRT use Characteristics at Oakdell Pharmacy

All women received progesterone (P4) as monotherapy (43%) or in combination with estrogen (57%). Few baseline characteristics differed between women on P4 monotherapy and estrogen + P4 combination therapy (Table 2). Women initiated on P4 monotherapy were younger (50 vs. 53 years, p < 0.01), less likely to be hysterectomized (27% vs. 38%, p = 0.02) and oophorectomized (21% vs. 32%, p = 0.04), and more likely to have a history of hypertension (17% vs. 9%, p = 0.04).

**Table 2:** Baseline Characteristics

Demographic	Estrogen + P4 (N = 170)	P4 (N = 126)	P-value (Estrogen + P4 vs. P4)
Age (yrs); mean (SD)	53 (7)	50 (10)	<0.01

Weight (lbs); mean (SD)	152 (32)	155 (32)	0.49

Comorbid Conditions; %			

Heart disease	2	1	0.46

Dyslipidemia	22	20	0.60

Hypertension	9	17	0.04

Diabetes	5	2	0.28

Cancer	2	3	0.67

Thyroid	14	13	0.72

Coagulopathies	2	1	0.28

Hysterectomized; %	38	27	0.02

Ovaries Removed; %	32	21	0.04

Tobacco use; %	8	9	0.95

Caffeine use; %	70	79	0.18

Alcohol use; %	54	52	0.51

Baseline CHT; %	8	9	0.74

Baseline BHRT; %	15	10	0.17

**Figure 1:**
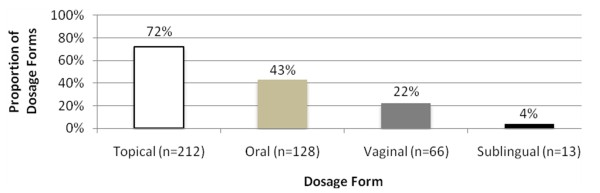
**Baseline BHRT Dosage Forms at Oakdell Pharmacy, n = 296**.

Various compounded BHRT dosage forms were utilized (Figure 1). Topical therapy was used most often (72%), followed by oral (43%), vaginal (23%), and sublingual (4%) therapy. Some women received multiple dosage forms simultaneously. Women receiving a single dosage form were initiated on topical therapy most often (40%), followed by oral (20%) and sublingual (2%). Women receiving combination dosage forms were initiated on topical+oral therapy most often (16%), followed by topical+vaginal therapy (12%), topical+oral+vaginal therapy (4%), oral+vaginal therapy (4%), and sublingual+oral therapy (2%). Women initiated on P4 monotherapy received more topical therapy (83% vs. 63%), and less oral (27% vs. 55%), vaginal (14% vs. 28%), and sublingual therapies (2% vs. 6%) than women initiated on estrogen + P4 combination therapy.

**Figure 2:**
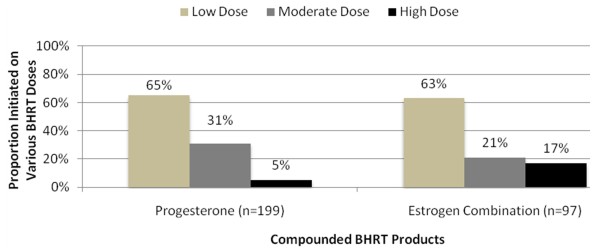
**Topical BHRT Doses at Baseline, n = 210**.

**Figure 3:**
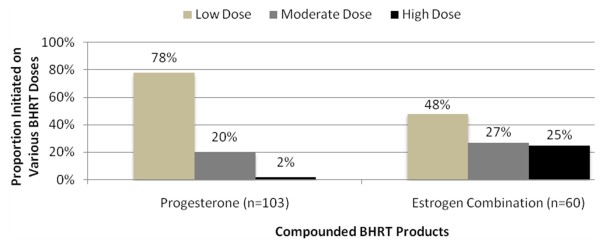
**Oral BHRT Doses at Baseline, N = 128**.

Therapy was generally initiated at low doses (Figure 2 and Figure 3). Low-dose progesterone monotherapy was initiated in 65% of patients receiving topical therapy and 78% of patients receiving oral therapy. Low-dose estrogen was initiated in 63% of patients receiving topical therapy and 48% of patients receiving oral therapy. Biest 50/50 (51%) and biest 80/20 (45%) were the most common topical estrogen combination therapies. Biest 50/50 (53%) and triest (35%) were the most common oral estrogen combination therapies. These estrogen combination therapies were also frequently initiated at low doses.

### BHRT Effectiveness

**Figure 4:**
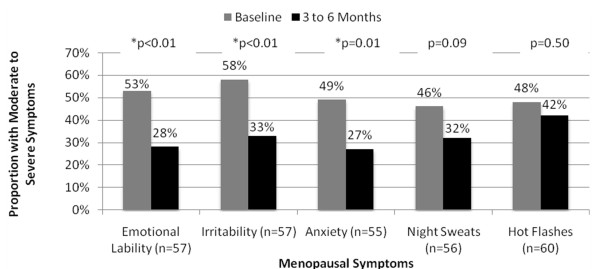
**Effectiveness of Compounded BHRT to Alleviate Moderate to Severe Menopausal Symptoms**.

Women initiated on compounded BHRT experienced significant reductions in moderate to severe mood symptoms within 3 to 6 months (Figure 4). Overall, women experienced a 25% decrease in emotional lability (53% vs. 28%, p < 0.01), a 25% decrease in irritability (58% vs. 33%, p < 0.01), and a 22% reduction in anxiety (49% vs. 27%, p = 0.01).

Women receiving compounded BHRT also experienced non-significant reductions in vasomotor symptoms within 3 to 6 months of therapy. Night sweats were reduced by 14% (46% vs. 32%, p = 0.09) and hot flashes were reduced by 6% (48% vs. 42%, p = 0.5) (Figure 4).

**Figure 5:**
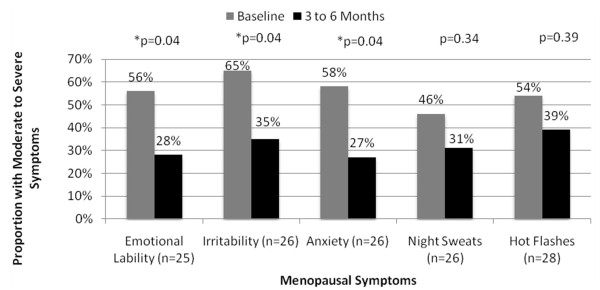
**Effectiveness of P4 Monotherapy to Alleviate Moderate to Severe Menopausal Symptoms**.

**Figure 6:**
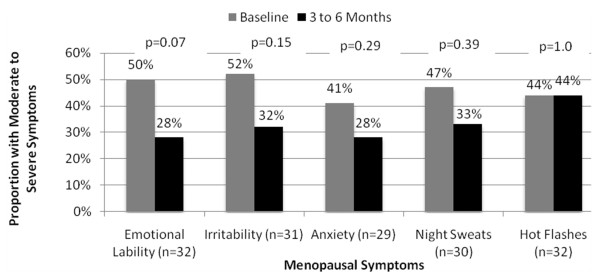
**Effectiveness of Estrogen + P4 Therapy to Alleviate Moderate to Severe Menopausal Symptoms**.

Women initiated on P4 monotherapy generally experienced a greater reduction in moderate to severe mood symptoms within 3 to 6 months compared to women initiated on estrogen + P4 combination therapy (Figure 5 and Figure 6). Women receiving P4 monotherapy experienced a 28% reduction in emotional lability (56% vs. 28%, p = 0.04), a 30% decrease in irritability (65% vs. 35%, p = 0.04), and a 31% reduction in anxiety (58% vs. 27%, p = 0.04). Although non-significant, women initiated on estrogen + P4 combination therapy experienced a 22% reduction in emotional lability (50% vs. 28%, p = 0.07), a 20% decrease in irritability (52% vs. 32%, p = 0.15), and a 13% reduction in anxiety (41% vs. 28%, p = 0.29). Moderate to severe night sweats decreased by 15% in women who received P4 monotherapy (46% vs. 31%, p = 0.34) and 14% in women who received estrogen + P4 combination therapy (47% vs. 33%, p = 0.39). Hot flashes were reduced by 15% with P4 monotherapy (54% vs. 39%, p = 0.39) and no improvements were seen with estrogen + P4 combination therapy (44% vs. 44%, p = 1.0).

**Figure 7:**
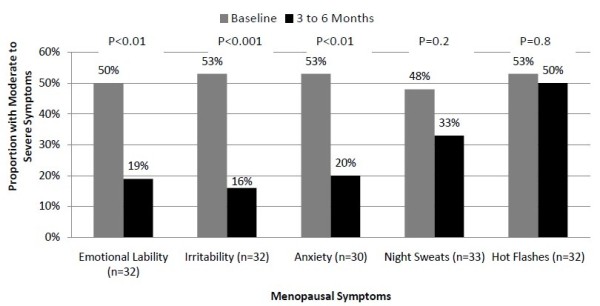
**Effectiveness of Compounded BHRT in Women Ages 40 - 70 years**.

We also performed a subgroup analysis to determine the effectiveness of compounded BHRT in women between the ages of 40 and 70 years (Figure 7). This age group more closely represents those women who are more likely to be affected by menopausal symptoms. The results seen were similar to those in the primary analysis. There was a 3% decrease in hot flashes (53% vs. 50%, p = 0.79) and a 15% decrease in night sweats (48% vs. 33%, p = 0.2). These women also experienced a 37% reduction in irritability (53% vs. 16%, p < 0.001), a 33% reduction in anxiety (53% vs. 20%, p < 0.01), and a 31% decrease in emotional lability (50% vs. 19%, p < 0.01).

### BHRT Safety

From this cohort of 296 women initiated on compounded BHRT, 62 (21%) had documented follow-up regarding myocardial infarction and breast cancer. These 62 women had an average follow-up of 1.9 years for a total of 117 person years. During this timeframe, no woman initiated on compounded BHRT experienced a myocardial infarction (MI) or breast cancer.

## Discussion

	This study confirms that compounded BHRT is effective for improving mood symptoms. We were unable to draw firm conclusions regarding the utility of compounded BHRT for vasomotor symptoms, but the observed trends are encouraging. Similar trends were seen in the subgroup analysis of women between the ages of 40 and 70 years.

This is the first time "compounded" BHRT therapy has been evaluated, but there are several studies that confirm the effectiveness of "manufactured" BHRT. Trials have shown estrone (E1) and estradiol (E2) to be effective for reducing menopausal symptoms as monotherapy or combination therapy, but trials have not been conducted to evaluate the effectiveness of estriol (E3).

The effectiveness of E1 for reducing the severity of menopausal symptoms has been observed. Takahashi [7] conducted an open label trial in 53 women receiving 2 mg E1 for 12 months. Menopausal relief was evaluated using the Kupperman index (KI) for severity. Women who underwent natural menopause experienced a 50% reduction in their KI score (p < 0.01), whereas women who underwent surgical menopause experienced an 80% reduction in their score (p < 0.01). The KI is a numerical conversion index of 11 menopausal symptoms (including vasomotor and mood symptoms) designed to measure total menopausal relief. Although individual symptoms were not evaluated, E1 was shown to be effective for relief of composite menopausal symptoms.

Large randomized controlled trials (RCTs) have documented E2's effectiveness for relieving menopausal symptoms [8,9]. Simon and colleagues [8] conducted a RCT in 484 women randomized to receive 0.87 grams/day (0.52 mg E2), 1.7 grams/day (1.02 mg E2), or 2.6 grams/day of E2 gel (1.56 mg E2) for 12 weeks. Within 3 to 5 weeks, these women experienced a reduction in hot flash severity (p < 0.01) and a reduction in hot flash rate by at least 7 hot flashes/day (p < 0.001). Additionally, the 1.7 grams/day E2 (1.02 mg E2) improved patient's Utian Quality of Life emotional score (0.9 vs. 0.2, p < 0.05). Similar to our method of evaluating severity, women reported hot flashes as severity ratings, yet limited numerical data were available to evaluate the extent of vasomotor symptom reduction. We acknowledge that the dose of E2 in our study was lower than most commercially available products. This may be one reason our study did not find a significant reduction in vasomotor symptoms with these compounded BHRT products.

Additionally, E1 and E3 combination therapy has been shown to be effective for reducing menopausal symptoms. Padwick and colleagues [10] conducted a 6 month RCT of 20 women randomized to receive oral E2 (2 mg) or oral E1 (1 mg) + E2 (2 mg). This study evaluated menopausal symptom relief using graphic rating scales. Women randomized to receive oral E2 (2 mg) experienced a significant reduction in hot flashes (62%, p < 0.01) and night sweats (77%, p < 0.01), and non-significant improvements in anxiety (26%), irritability (18%), and emotional lability (5%) (p values not given). Women receiving oral E1 (1 mg) + E2 (2 mg) experienced significant reductions in hot flashes (41%, p < 0.01), night sweats (56%, p < 0.05), and anxiety (44%, p < 0.01), and non-significant improvements in irritability (32%) and emotional lability (5%). Despite use of a different method to evaluate symptom relief, this study provides additional data that supports the use of combination estrogens for reducing vasomotor and mood symptoms.

Although estrogens are generally regarded as the therapeutic hormones in hormone replacement therapy [11], P4 monotherapy has also been shown to be effective in treating menopausal symptoms [12-14]. Leonetti and colleagues [12] conducted a single-center RCT of 102 women within 5 years of menopause who were randomized to receive P4 monotherapy (20 mg) or placebo. Significant improvements or resolution of vasomotor symptoms were seen at 4 months in P4-treated patients compared to placebo (83% vs 19%; p < 0.001). Two other RCTs have documented non-significant improvements in menopausal symptoms [13,14]. Wren and colleagues [13] evaluated 80 women randomized to receive topical P4 monotherapy (32 mg) or placebo. A reduction in vasomotor (-1.0 vs. 0; p = 0.07) and anxiety (-1.0 vs. 0; p = 0.10) symptoms was reported at 12 weeks. Another RCT conducted by Benster and colleagues [14] randomized 223 women to receive P4 monotherapy (5 mg, 20 mg, 40 mg, and 60 mg) or placebo. The investigators observed a reduction in vasomotor symptoms for P4 5 mg (-0.4; p = 0.22), 20 mg (-0.4; p = 0.23), 40 mg (-0.6; p = 0.06), and 60 mg (-0.4; p = 0.23). These trials utilized the Greene Climacteric Scale, in which patients self-rated their symptom severity (i.e. absent, mild, moderate, and severe). This approach is similar to our study.

Manufactured E2 + P4 combination therapy has also demonstrated effectiveness in reducing menopausal symptoms. Vashisht and colleagues [15] conducted an open label 48-week study in 41 women with the secondary objective of determining improvements in menopausal symptoms. Treatment with E2 gel (1 mg) + P4 cream (40 mg) applied daily significantly reduced moderate to severe vasomotor and anxiety symptoms at 24 weeks (p < 0.05). Anxiety was further reduced at 48 weeks of therapy when compared to the reduction at 24 weeks of therapy (p < 0.05). Similar to P4 monotherapy trials, the Greene Climacteric Scale was utilized to evaluate mood and vasomotor symptom improvement.

Collectively, these findings support the effectiveness of BHRT in reducing menopausal symptoms. Furthermore, this study provides some of the first clinical evidence in support of "compounded" BHRT.

### BHRT Safety

Large RCTs by the Women's Health Initiative (WHI) and Heart and Estrogen/Progestin Replacement Study (HERS) have documented deleterious effects associated with CHT in postmenopausal women. Woman randomized to receive CE + MPA combination therapy experienced a 1.47 and 1.32 fold increase in non-fatal MI in the HERS and WHI studies, respectively [16,17]. The WHI also demonstrated a 1.26 fold increase in breast cancer in patients receiving CE + MPA [16]. There are currently no trials that evaluate BHRT risk for MI and breast cancer. This study only had 117 person-years of follow-up and none of these women experienced an MI or breast cancer; however, the study lacks a sufficient sample size to make firm statements about the safety of compounded BHRT.

It is noteworthy that the Postmenopausal Estrogen/Progestin Interventions (PEPI) trial, Gerhard trial, and Estrogen in Prevention of Atherosclerosis (EPAT) trials have all three demonstrated that BHRT improves cardiovascular surrogate markers. Women in the PEPI trial who received CE + cyclic P4 experienced a decreased in LDL, increase in HDL, decrease in fibrinogen levels, and decrease in fasting glucose compared to those women on placebo [18]. Gerhard and colleagues [19] demonstrated that women who received transdermal E2 + cyclical vaginal P4 experienced improved vascular reactivity and an 8.4% reduction in LDL compared to placebo. Finally, the EPAT trial demonstrated that women who received oral E2 compared to placebo experienced a decrease in carotid intima thickness progression, increase in HDL, decrease in LDL, and decrease in HbA1c [20]. Based on the favorable effects on cardiovascular surrogate markers, BHRT may eventually prove to be cardio-neutral or even cardioprotective.

Smaller RCTs provide encouraging results regarding the impact of BHRT on breast cancer. Two RCTs by Chang and colleagues [21] and Foidart and colleagues [22] demonstrated that women receiving topical P4 experienced a reduction in breast epithelial proliferative markers via reductions in mitotic divisions and proliferating cell nuclear antigen (PCNA) labeling index % compared to placebo. These studies support the belief that P4 may prevent breast epithelial hyperplasia. A large observational study demonstrated that women who used E3 or estrogen + P4 combination therapy were not at increased risk of breast cancer as compared to women who had never used HRT [23].

### Limitations

This study has strengths and limitations. First, these six pharmacies were located in one geographical region; therefore, these findings may not be generalized to all settings. We detected a significant improvement in mood symptoms; however, the sample size was insufficient to assess the safety of compounded BHRT or its impact on vasomotor symptoms. Our method of obtaining effectiveness data also lends itself to potential rater bias. Patients might have attempted to please the health care provider by reporting improvement in menopausal symptoms. Additionally, the rating scale utilized in the majority of "manufactured" BHRT clinical trials differed from this study. The Greene Climacteric Scale and The Kupperman index may be more effective for evaluating menopausal symptom outcomes with HRT. Also, this study did not evaluate changes in regimens from baseline to follow-up (3 to 6 months); therefore, improvements in menopausal symptoms were attributed to the initial regimen women received. Finally, the results of this study may be more generalizable to hysterectomized women and those with previous or current BHRT or CHT use since more women were included in this study with those characteristics compared to those women excluded.

## Conclusions

Our study is one of the first to provide clinical evidence for the effectiveness of compounded BHRT. Given the deleterious effects of CHT observed in the WHI and HERS trials, these data are of great value. Our study provides clear evidence that compounded BHRT is effective for reducing menopausal mood symptoms. Larger studies are needed to examine the impact of compounded BHRT on vasomotor symptoms, myocardial infarction, and breast cancer.

## Competing interests

ADR and JJC are employees of Oakdell Pharmacy, Inc. All other authors declare that they have no competing interests.

## Authors' contributions

ADR had full access to study data and was the primary person involved in the study design, data collection, data analysis, data interpretation, and manuscript drafting. CRF and KRD were involved in the study design, data analysis, data interpretation, original manuscript drafting, and manuscript revisions. JCB and JJC were involved in the study design and manuscript editing. All authors have read and approved the final manuscript.

## Pre-publication history

The pre-publication history for this paper can be accessed here:

http://www.biomedcentral.com/1472-6874/11/27/prepub
